# Geometry-Aware Cell Detection with Deep Learning

**DOI:** 10.1128/mSystems.00840-19

**Published:** 2020-02-04

**Authors:** Hao Jiang, Sen Li, Weihuang Liu, Hongjin Zheng, Jinghao Liu, Yang Zhang

**Affiliations:** a College of Science, Harbin Institute of Technology, Shenzhen, China; University of California San Diego

**Keywords:** ExtremeNet, adjacency spectrum, cell detection, geometry aware, microscopic image, protozoa

## Abstract

Analyzing cells and tissues under a microscope is a cornerstone of biological research and clinical practice. However, the challenge faced by conventional microscopy image analysis is the fact that cell recognition through a microscope is still time-consuming and lacks both accuracy and consistency. Despite enormous progress in computer-aided microscopy cell detection, especially with recent deep-learning-based techniques, it is still difficult to translate an established method directly to a new cell target without extensive modification. The morphology of a cell is complex and highly varied, but it has long been known that cells show a nonrandom geometrical order in which a distinct and defined shape can be formed in a given type of cell. Thus, we have proposed a geometry-aware deep-learning method, geometric-feature spectrum ExtremeNet (GFS-ExtremeNet), for cell detection. GFS-ExtremeNet is built on the framework of ExtremeNet with a collection of geometric features, resulting in the accurate detection of any given cell target. We obtained promising detection results with microscopic images of publicly available mammalian cell nuclei and newly collected protozoa, whose cell shapes and sizes varied. Even more striking, our method was able to detect unicellular parasites within red blood cells without misdiagnosis of each other.

**IMPORTANCE** Automated diagnostic microscopy powered by deep learning is useful, particularly in rural areas. However, there is no general method for object detection of different cells. In this study, we developed GFS-ExtremeNet, a geometry-aware deep-learning method which is based on the detection of four extreme key points for each object (topmost, bottommost, rightmost, and leftmost) and its center point. A postprocessing step, namely, adjacency spectrum, was employed to measure whether the distances between the key points were below a certain threshold for a particular cell candidate. Our newly proposed geometry-aware deep-learning method outperformed other conventional object detection methods and could be applied to any type of cell with a certain geometrical order. Our GFS-ExtremeNet approach opens a new window for the development of an automated cell detection system.

## Abstract (precis)

Automated diagnostic microscopy powered by deep learning is useful, particularly in rural areas. However, there is no general method for object detection of different cells. In this study, we developed GFS-ExtremeNet, a geometry-aware deep-learning method which is based on the detection of four extreme key points for each object (topmost, bottommost, rightmost, and leftmost) and its center point. A postprocessing step, namely, adjacency spectrum, was employed to measure whether the distances between the key points were below a certain threshold for a particular cell candidate. Our newly proposed geometry-aware deep-learning method outperformed other conventional object detection methods and could be applied to any type of cell with a certain geometrical order. Our GFS-ExtremeNet approach opens a new window for the development of an automated cell detection system.

## INTRODUCTION

Automatic detection of different types of cells in microscopy images are of significant interest to a wide range of biological research and clinical practices. Therefore, several computer-aided cell detection methods, ranging from decision trees to deep-learning-based techniques, have been proposed (1–4). In the early years, many efforts were devoted to extracting handcrafted features for cell detection and classification (1, 2). However, they faced several challenges, including incomplete feature representation, the high complexity of the detection methods, and low accuracies of detection. New methods using a deep-learning architecture are able to extract the depth features of images more comprehensively. Prior knowledge of the features to be selected is unnecessary; thus, the accuracy of detection is significantly improved by avoiding the misrepresentation of important features. However, the target is essentially detected as a point rather than a complex, ignoring details such as size and shape (3–5).

Object detection using deep learning is revolutionizing various areas, including entertainment (6), surveillance (7), and even self-driving automobiles (8). Deep-learning frameworks for object detection can be grouped into two different approaches: one-stage detection (e.g., single-shot detection [SSD], you only look once [YOLO], CornerNet, and ExtremeNet [9–12]) and two-stage detection (e.g., regional convolutional neural network [R-CNN] family, including R-CNN, Fast R-CNN, Faster R-CNN, and Mask R-CNN [13–16]). It is known that the two-stage approach is superior in detection and positioning accuracy, but the one-stage approach is faster in detection speed (12). Among the one-stage frameworks, ExtremeNet is considered to be the best model in terms of performance and accuracy (12). This approach employs an hourglass network as its backbone network for key-point detection, including extreme points and center points, and key points are combined with the method of exhaustion (12, 17).

For microscopic cell detection, ExtremeNet is prone to a combination error in the case of multiple cell targets and overlapping targets because the geometric correlation between every two key points is not considered in the key-point combination.

Recently, some implementations that include morphological features indicating the shapes of target cells in the deep-learning models were reported. Tofighi et al. (18) developed shape priors with convolutional neural networks (SP-CNN) by learning the shapes of cells. Falk et al. (5) reported a universal network (U-Net) using deep learning for cell counting, detection, and even shape measurements. Nevertheless, these implementations are still inept for new cell targets with a variety of cell shapes and sizes, resulting in imperfect localization and detection (3).

**FIG 1:**
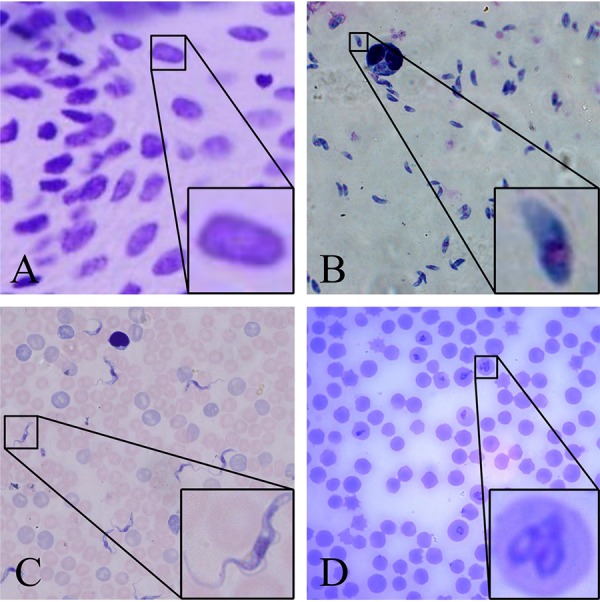
Examples of typical cell images under the microscope. The boxes frame a few targets that need to be detected and magnified. (A) Nucleus; (B) *Toxoplasma*; (C) *Trypanosoma*; (D) *Babesia*.

It has long been recognized that cells show distinct and defined geometric order of a given type. Cells are generally round, but the cells of some unicellular organisms show great variations in terms of shape and size. For example, protozoa and single-celled eukaryotes, including some parasites, exhibit morphological features distinct from those of mammalian cells (Fig. 1).

Herein, we propose a simple and efficient geometry-aware deep-learning approach for cell detection. By converting the thought that each cell has its own unique geometric order, we redesigned the ExtremeNet framework by incorporating a geometric-feature spectrum. We refer to this network as the geometric-feature spectrum ExtremeNet (GFS-ExtremeNet), which introduces geometric information into the key-point combination process to achieve improved accuracy in cell detection.

To illustrate this improved performance, we show the high accuracy and effectiveness of our GFS-ExtremeNet approach with the publicly available data set of mammalian cell nuclei (Fig. 1A) by outperforming other state-of-the-art alternatives (Table 1). In addition, we demonstrate the generality of our GFS-ExtremeNet model in solving complicated microscopic cell identification tasks with three newly collected unicellular parasitic protozoa, namely, *Toxoplasma*, *Babesia*, and *Trypanosoma* parasites. *Toxoplasma* is a single-cell protozoan parasite capable of infecting all warm-blooded animals as well as one-third of the world’s human population (19–21). The name *Toxoplasma* is derived from the Greek word *toxon*, meaning arc or bow shaped, in reference to the unique crescent shape of the parasite (Fig. 1B). *Trypanosoma* parasite, a unicellular protozoan, infects wild and domesticated animals and humans. In humans, *Trypanosoma* parasite is a causative agent of Chagas disease in Latin America and African sleeping sickness in sub-Saharan Africa (22), and these diseases are some of the most severe public health problems in these developing countries. In the blood plasma of patients, a *Trypanosoma* parasite forms a thin, flattened, and spindle-shaped body (Fig. 1C). *Babesia*, an intraerythrocytic protozoan parasite, is responsible for a malaria-like illness that imposes a significant health burden on animals and occasionally humans worldwide (23, 24). The *Babesia* parasites are characteristically pear shaped (Fig. 1D), and sometimes an irregularly shaped form may also be found within red blood cells (RBCs). Microscopy is the most commonly used method in the diagnosis and analysis of these parasites from infection samples. Identifying and analyzing the parasite cells accurately and efficiently can help reduce the disease burden, especially in areas with limited resources.

**TABLE 1:** Results of GFS-ExtremeNet and baselines for the nucleus data set

Approach	Method	Backbone	AP(0.5:0.95)	AP75	AP50
Two stage	Faster R-CNN	ResNet-101	21.45	23.96	35.89
	Mask R-CNN	ResNet-101	45.20	51.82	67.20
One stage	SSD	ResNet-101	14.32	11.42	32.54
	YOLOv3	DarkNet-53	38.68	33.46	76.76
	CornerNet	Hourglass-104	74.18	79.23	83.46
	GFS-CornerNet	Hourglass-104	74.86	81.07	85.87
	ExtremeNet	Hourglass-104	76.66	85.84	88.41
	GFS-ExtremeNet	Hourglass-104	77.96	87.47	90.22

## RESULTS

### Comparison of different target detection models as the backbone network.

To compare various common target detection algorithms, we tested two-stage algorithms, namely, Faster R-CNN and Mask R-CNN, and one-stage algorithms, namely, SSD, YOLOv3, CornerNet, and ExtremeNet, on the nucleus data set. As shown in Table 1, ExtremeNet outperformed all other backbone methods in its AP(0.5:0.95), AP75, and AP50. AP(0.5:0.95) corresponds to the average precision (AP) for the intersection-over-union (IoU) threshold from 0.5 to 0.95, with a step size of 0.05. The IoU threshold was set to 0.5 (AP50) and 0.75 (AP75).

**FIG 2:**
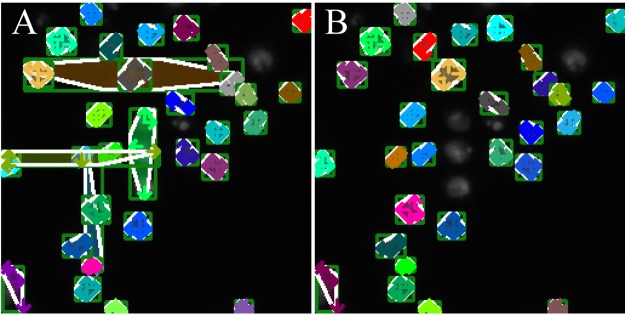
GFS-ExtremeNet’s role in microscopic image detection. (A) ExtremeNet’s poor performance with microscopic images; (B) GFS-ExtremeNet’s improved detection result.

Although ExtremeNet has the best performance in the nucleus data set, this algorithm cannot be used directly for microscopic images. Because of the complexity of a microscope image, the distributions of cells are globally sparse and locally dense, with overlapping and juxtaposition. As a result, in the combination process of extreme points, the extreme point of a given target might be mistakenly paired with the adjacent extreme point of the other target, resulting in an incorrect recognition result (Fig. 2A). The reason for this erroneous result is that the algorithm generates only extreme points and center points without consideration for the relationship between the points. As a result, the points that have been combined are likely to be combined with other points again, resulting in an incorrect combination result. To overcome these drawbacks, we introduced a geometry-aware software into ExtremeNet by using the adjacency spectrum, namely, GFS-ExtremeNet. The improved detection results through GFS-ExtremeNet are shown in Table 1 and Fig. 2.

### GFS-ExtremeNet.

It has long been recognized that cells show distinct and defined geometric order of a given type. Cells are generally round, but some unicellular organism cells show great variations in terms of shape and size.

**FIG 3:**
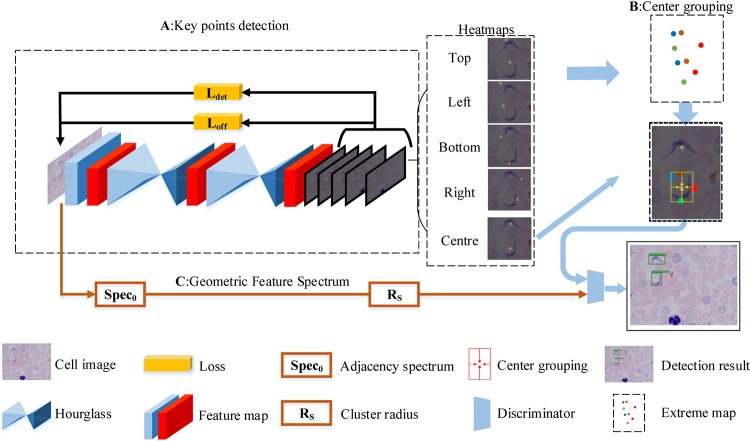
Schematic representation of the GFS-ExtremeNet algorithm in microscopic-image detection. Overall, this geometry-aware approach is divided into two parts. (A) The first part is using the hourglass network to extract the extreme and the center points of the target and generating heatmaps for those points. In addition, the relationship of the extreme points is measured and passed to the second stage in forming the adjacency spectrum. (B) The second part is the feature combination, mainly center grouping. (C) After the geometric relationship is combined, the adjacency spectrum is then used for verification to form the detection results.

By considering the thought that each cell has its own unique geometric order, we redesigned ExtremeNet by incorporating a geometric feature, the adjacency spectrum (Fig. 3). We evaluated our proposed GFS-ExtremeNet method on the same publicly available nucleus data set. As can be seen from the results in Table 1, our GFS-ExtremeNet algorithm outperformed ExtremeNet.

In order to evaluate the effectiveness and generalization of the geometry-aware approach, we also tested the performance of the geometric-feature spectrum on CornerNet, which is another commonly used object detection framework. As shown in the Table 1, the performance of GFS-CornerNet was substantially reduced compared to that of GFS-ExtremeNet but better than that of CornerNet. These results illustrated the superiority of ExtremeNet and the importance of the geometric-feature spectrum.

### Detection performance with parasite microscopic images.

**FIG 4:**
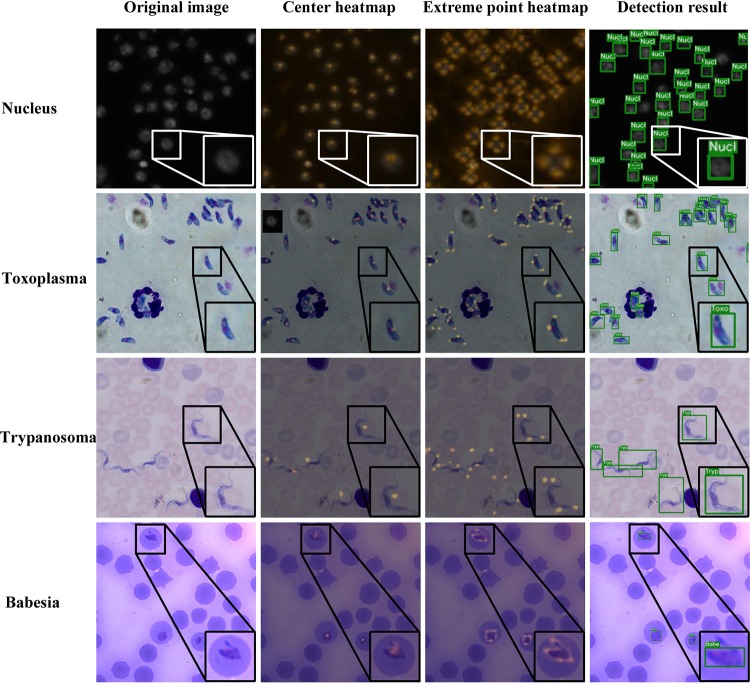
Detection results obtained by GFS-ExtremeNet. Examples of nuclei (Nucl), *Toxoplasma* (Toxo), *Trypanosoma* (Tryp), and *Babesia* (Babe) parasites are presented. The first-column images are the original images to be detected, the second-column images are the center-point heatmaps, and the third-column images are extreme-point heatmaps. The fourth-column images are the final test detection result, where the target is identified by the box frame, with the name and score of the target shown.

To investigate the generalizability of our system to the detection of different microscopic cell images, we conducted the same deep-learning framework analysis on several unicellular protozoon microscopic images, including banana-shaped *Toxoplasma*, spindle-shaped *Trypanosoma* parasite, and pear-shaped *Babesia*. Some examples of those microscopic images are shown in Fig. 4. From the first row of results in Fig. 4, we prove that our model performs satisfactorily in detecting nuclei, missing very few targets. From the second row of results, our GFS-ExtremeNet can successfully detect numerous overlapping and aggregated forms of *Toxoplasma* in the stained images, distinguishing them from host cells and debris. *Trypanosoma* parasite has an elongated and flattened leaf-like body with a flagellum on its end. The use of this parasite is to prove that our model can obtain acceptable results in cells with complicated shapes. As a result, the GFS-ExtremeNet algorithm detected all *Trypanosoma* parasites, not RBCs. All three types of cells mentioned above are isolated ones, which leads us to wonder if our model can detect organisms properly in even more difficult situations. The parasite *Babesia*, like the malaria agent, can reside and replicate within RBCs, representing a picture-in-a-picture situation. As shown in the last line of Fig. 4, a high accuracy of detection was obtained for this intraerythrocytic parasite.

In comparison, we measured the AP(0.5:0.95), AP75, and AP50 values of different parasitic targets, including *Toxoplasma*, *Trypanosoma*, and *Babesia*, with both GFS-ExtremeNet and ExtremeNet (Table 2). As shown in Table 2, as with the nucleus data set, GFS-ExtremeNet outperformed ExtremeNet for all three parasites targets, indicating the irreplaceable role of the geometric-feature spectrum in cell detection.

**TABLE 2:** Results of GFS-ExtremeNet and ExtremeNet on parasite microscopic images^*a*^

Parasite	Model	AP(0.5:0.95)	AP75	AP50
*Babesia*	ExtremeNet	35.87	23.18	72.90
	GFS-ExtremeNet	37.14	24.55	74.16
*Trypanosoma*	ExtremeNet	58.30	68.23	92.00
	GFS-ExtremeNet	58.72	68.41	95.02
*Toxoplasma*	ExtremeNet	30.91	22.60	68.47
	GFS-ExtremeNet	32.70	23.50	69.77

a The performances of GFS-ExtremeNet (with geometric-feature spectrum) and ExtremeNet (without geometric-feature spectrum) with three parasites targets were compared.

**FIG 5:**
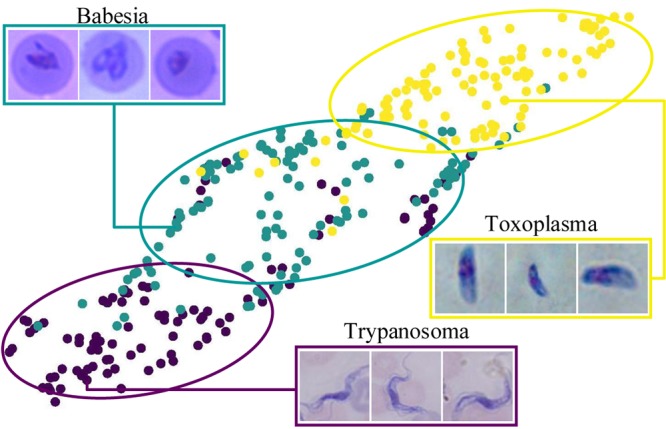
t-SNE plot of GFS-ExtremeNet. t-SNE provides a method to evaluate and refine the clustering of different parasite cells’ images. Data points are colored according to their labels.

To visualize how capable the GFS-ExtremeNet model is in distinguishing between different parasites, we deployed a two-dimensional (2D) *t*-distributed stochastic neighbor embedding (t-SNE) plot to show cluster performance (Fig. 5). t-SNE can be used to visualize high-dimensional data in 2D, maintaining local structures. The t-SNE plot shows three separated distribution clusters, indicating that GFS-ExtremeNet can correctly distinguish different parasites.

The practicality of the model is largely determined by how well it will do when asked to make new predictions for data that it has not already seen. To avoid the bias of the images acquired, we gathered 10 different trypanosome images from the Internet, representing the data style in a variety of different situations. Our GFS-ExtremeNet model was able to achieve an average of 94.4% recognition accuracy with these new images.

### In comparison with biologists.

**FIG 6:**
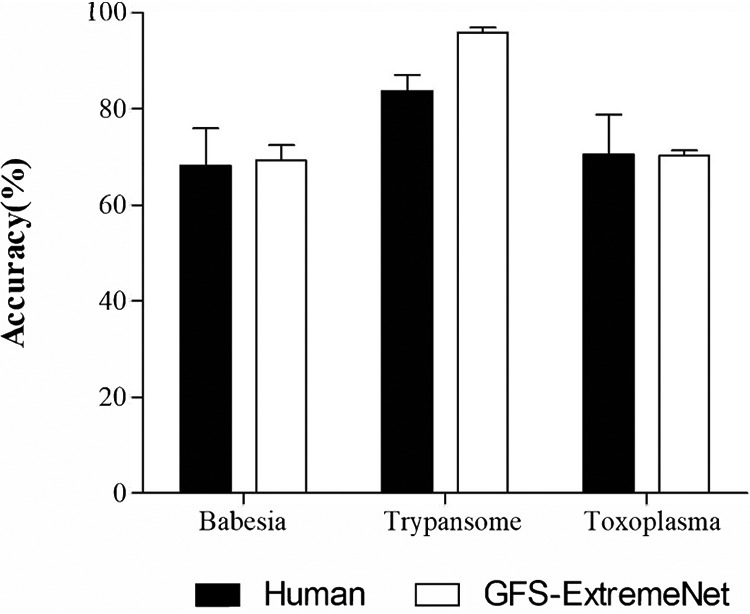
The accuracies of humans were compared to those of GFS-ExtremeNet. The performance of GFS-ExtremeNet is similar to or better than that of humans.

To compare our GFS-ExtremeNet model with human biologists, the participants and the machine were provided with each of the 130 parasite images. Four experienced annotators commented on the images to identify the parasites independently (Fig. 6). Close analysis of the results reveals that the GFS-ExtremeNet model achieved 69.26% ± 3.68%, 70.22% ± 1.31%, and 95.89% ± 1.18% average accuracies for *Babesia*, *Toxoplasma*, and *Trypanosoma*, respectively, while humans achieved 68.25% ± 8.92%, 70.50% ± 9.57%, and 83.75% ± 3.86% average accuracies. As seen from this result, in terms of accuracy, the performance of GFS-ExtremeNet is similar to or better than that of humans. Moreover, the high error bar for humans indicates the variable performance of humans. It cannot be easy for inexperienced clinicians to distinguish between different cell targets and even between cells and artifacts (such as stain or platelet debris) by the physical examination of slides alone. Therefore, the detection accuracy highly depends on how well trained and experienced the professionals are. The deep-learning system may potentially aid clinicians in these examinations by providing automated, accurate, and timely testing.

### Discussion and conclusions.

Our study is the first to investigate a cell recognition task with the use of a geometric-feature spectrum as well as the first to propose a geometry-aware deep-learning approach. Cell shape and size can vary considerably, making an algorithm for reliable detection difficult. Parasites can live inside host cells and their shapes can overlap erythrocytes to make recognition even more difficult. Very few of the previous works considered the importance of shape in cell target detection. To address these challenges, we proposed the GFS-ExtremeNet model, enabling systematic learning of the features from a determined area within extreme points. Through experiments on publicly available and self-obtained microscopic cell images, we have successfully demonstrated that our GFS-ExtremeNet model can detect multiple cell types, including *Babesia*, *Toxoplasma* and *Trypanosoma* parasites, with high accuracy. However, the labeling of specific extreme points that best reflect the geometric features of a target of interest is a prerequisite for new-target training. It may limit the use of this method by a nonprofessional developer. As a result, the accuracy is highly dependent on the labeling of the extreme point in the innate area of data training. In the future, an automatic extreme-point detection method which will be able to reduce the time needed for labeling and be broadly applicable by the general public and inexperienced clinicians will be developed.

Moreover, the internal rules in our model for feature selection within the extreme boxes are not well understood. Traits such as color and texture might be also important for the deep-learning model to recognize the target. Therefore, to build a more reliable model, images from other testing scenarios need to be learned by the model.

Our model can be applied to any microscope image targets with certain geometric orders, showing promising and broader application potential for microscopic image analysis. This is immensely helpful in the development of an automated cell detection system with improved efficiency and a reduced error rate.

## MATERIALS AND METHODS

### Data set.

Four different microscopic image data sets, including images from nuclei, *Toxoplasma*, *Trypanosoma*, and *Babesia*, were used in this study. For nuclei, we used a publicly available data set to verify and compare the performance of our model with those of alternative models. A total of 670 whole-slide images (WSIs) with 29,461 nuclei were used, and images were split into train, test, and verification sets, with a ratio of 8:1:1. With complete annotations for object segmentation masks made, we found the extreme points in the four directions of the mask and used them as our annotations of targets.

The other three sets of parasite images were acquired with a bright-field light microscope (Olympus IX53) with 100× oil immersion objectives. In total, we collected 261 *Toxoplasma*, 480 *Trypanosoma*, and 567 *Babesia* WSIs. We used LabelMe (25) to obtain annotations of these parasites, and we then marked the four extreme points of each target.

### Deep-learning methods for object detection.

Deep-learning methods for object detection can be grouped by two different approaches: one-stage detection and two-stage detection. Faster R-CNN and Mask R-CNN, two representative algorithms for two-stage detectors, are selected in this study. YOLO, SSD, CornerNet, and ExtremeNet, main representatives of one-stage algorithms, were also evaluated. The detailed algorithms and model setting followed those in previously described papers (9–12).

### ExtremeNet.

ExtremeNet, one of the most state-of-art algorithms mainly converts the detection of key points to extreme points in four directions and a central point. In the first part, four extreme-point heatmaps and one center-point heatmap are detected by the standard key-point estimation network, the hourglass network (usually used in the human pose estimation field). In the second part, the extracted key points are combined through pure geometric theory, and a set of extreme points corresponding to a detection target are formed. The detailed algorithm is divided into two steps, key-point detection and center grouping (12). In key-point detection, the algorithm transforms the problem of target detection into key-point estimation, avoiding regional classification and feature learning. A multichannel heatmap was predicted using the fully convolutional encoder-decoder network. Using the hourglass network as the backbone, each heatmap was weighted point by point logically, the purpose of which was to reduce the number of false-negative results around the ground truth. The loss of the improved version was used in the training network, as follows:

Ldet⁡=−1N∑i=1H∑j=1W(1−Y^ij)α log⁡(Y^ij)(1−Yij)β (Y^ij)α log⁡(1−Y^ij)if Yij=1 otherwise

where $L_{\text{det}}$ is the improved focal loss, H is the length of the microscopic image (represented by pixels), W is the width, and N is the number of targets in the image. The value of Y^ij is the predicted score of the location, 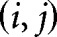, in the heatmap through the network, and $Y_{\mathrm{ij}}$ is the corresponding true value. Both α and β are hyper-parameters, equal to 2 and 4, respectively.

To improve the accuracy of target detection, a class-independent offset map was added to the algorithm to compensate for the resolution loss $\left(L_{1}\right)$ caused by the process of down-sampling. Smooth $L_{1}$ was used during offset map training 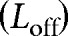, as follows:

$$L_{\text{off}}=\frac{1}{N}\overset{N}{\underset{k=1}{\sum }}SL_{1}[\Delta^{\left(\alpha \right)},\overset{\to }{x}/s-\left\lfloor \overset{\to }{x}/s\right\rfloor ]$$

where ${SL}_{1}$ is the smooth *L*_1_ loss, Δ^(α)^ is the key point offset, $\overset{\to }{x}$ is the coordinate of the key point, and s is the down-sampling factor.

Under the supervision of the above two losses, $L_{\text{det}}$ and 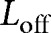, the network outputs four extreme-point heatmaps and one center map: Y^(c),Y^(t),Y^(l),Y^(b),Y^(r) ∈ (0,1)H×W.

As can be seen from the above $L_{\text{det}}$ and 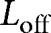, this key-point-based target detection algorithm has an advantage: a greatly reduce labeling time compared with those of other supervised methods. In the microscopic image, there is a large number of parasites with different shapes. The algorithm needs to manually mark only the four extreme points of each target. Because of the mentioned advantages, we used the hourglass network and the two losses of $L_{\text{det}}$ and $L_{\text{off}}$ in GFS-ExtremeNet.

In addition, we used the hourglass network as the backbone network. As used mainly in pose estimation, the basic network is a fully convolutional neural network. Given a single red, green, and blue (RGB) image, the network outputs the precise pixel position of the key points of the target using multiscale features to capture the spatial position of each joint point of the target. Its network structure is shaped like an hourglass, hence the name “hourglass network” (17).

For center grouping, ExtractPeak (12) first extracts all the extreme points in the heatmap, which are defined as the maximum values in the 3 by 3 sliding window, forming four sets of extreme points: 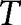, 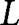, 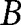, and 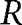. The second step is brute-force algorithm in which the center point of each extreme-point combination, 
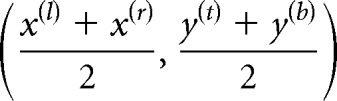
, is calculated. If the response at the corresponding position of the center map exceeds the present threshold, 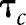, then this set of five points is taken as a temporary result because of its geometric ignorance between adjacent key points, and the score of the candidate combination is the average of the five corresponding points.

### Adjacency spectrum.

According to the shortcomings of ExtremeNet described above, from the perspective of graph theory, we introduced the adjacency spectrum into the key-point combination process of ExtremeNet to measure the geometric relationship of each key point of the target cell to the adjacency spectrum. The process of model building is as follows:

1. The hourglass network extracts extreme points in four directions, as follows: 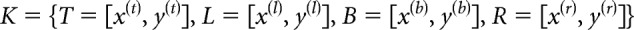, where 
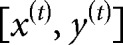
 is the top extreme point, 
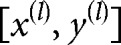
 is the left extreme point, 
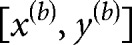
 is the bottom extreme point, and 
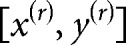
 is the right extreme point.
2. According to graph theory, the graph 
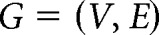
 is constructed by using the above four extreme points. 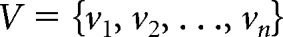 is the node set in graph 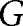, and 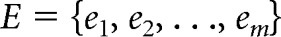 is the edge set formed by the connection of two adjacent nodes in 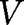. For example, $G_{\text{cell}}$ is the graph constructed by four extreme points of the cell. E has no directionality in our model, so G is an undirected graph.
3. The *n*th-order square matrix, 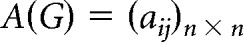, constructed based on 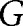, is called the adjacency matrix of graph 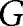 and is denoted by 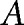, where
  $$a_{\mathrm{ij}}=\left\{ \begin{array}{l} \omega_{\mathrm{ij}}\\ 0 \end{array}\right.\begin{array}{c} \text{if there are connections between}\;v_{i}\;\text{and}\;v_{j}\\ \text{if there are no connections}\;v_{i}\;\text{and}\;v_{j} \end{array}.$$
  The weight, 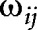, is expressed by the Euclidean distance, 
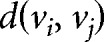
, between two points; that is, 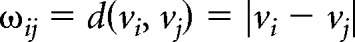. The adjacency matrix is denoted
  $$A(G)=\left(\begin{array}{cccc} a_{11} & a_{12} & \cdot \cdot \cdot & a_{1n}\\ a_{21} & a_{22} & \cdot \cdot \cdot & a_{2n}\\ \cdot \cdot \cdot & \cdot \cdot \cdot & \cdot \cdot \cdot & \cdot \cdot \cdot \\ a_{n1} & a_{n2} & \cdot \cdot \cdot & a_{\mathrm{nn}} \end{array}\right).$$
4. Calculate the adjacency spectrum, 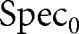, of graph G from adjacency matrix 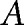. The characteristic polynomial corresponding to A is 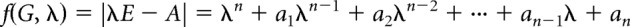, $a_{n}$ is the coefficient in the characteristic polynomial, and the calculated eigenvalues, λ of 
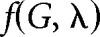
, form the adjacency spectrum, ${\text{Spec}}_{0}$ of 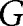, and the process is transformed into a solution of the characteristic equation 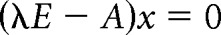; that is,
  $$\left|\lambda E-A\right|=\left|\begin{array}{cccc} \lambda -a_{11} & -a_{12} & \cdot \cdot \cdot & -a_{1n}\\ -a_{21} & \lambda -a_{22} & \cdot \cdot \cdot & -a_{1n}\\ \cdot \cdot \cdot & \cdot \cdot \cdot & \cdot \cdot \cdot & \cdot \cdot \cdot \\ -a_{m1} & -a_{11} & \cdot \cdot \cdot & \lambda -a_{\mathrm{mn}} \end{array}\right|=0,$$
  which solves for *n* complex roots, and 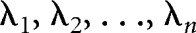 are the *n* eigenvalues of the adjacency matrix minus those of the adjacency spectrum, 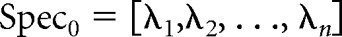.

### GFS-ExtremeNet.

**FIG 7:**
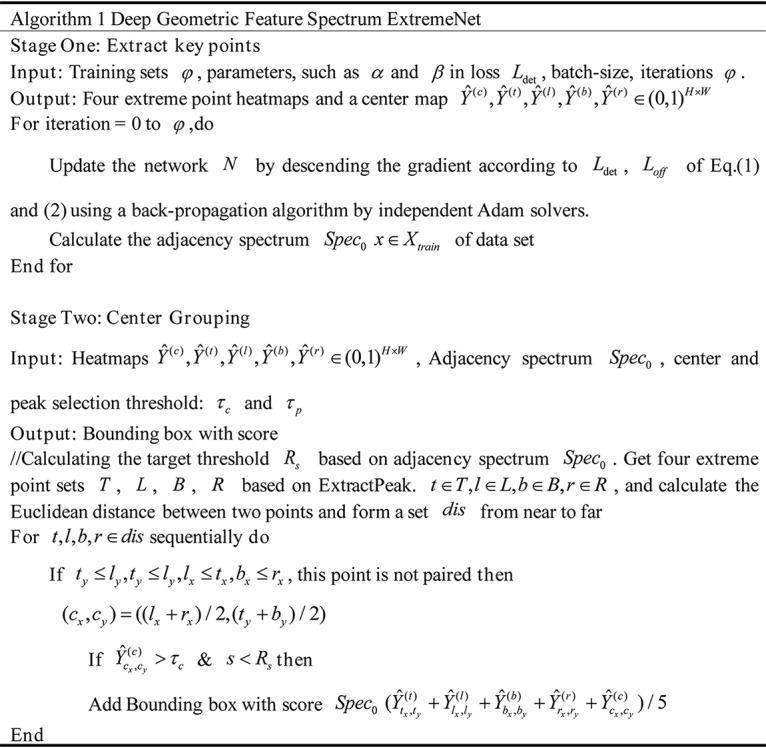
The algorithm of deep geometric-feature spectrum ExtremeNet (GFS-ExtremeNet).

The architecture of the GFS-ExtremeNet algorithm is based on the basis of ExtremeNet with the introduction of the adjacency spectrum. In the first stage, parameters such as α and β in loss 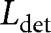, batch size, and the maximum number of iterations, 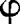, were optimized. After training the hourglass network with the losses of $L_{\text{det}}$ and 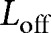, back-propagation, and the use of the Adam optimizer to update the parameters in network 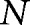, we obtained four extreme-point heatmaps and a center map: 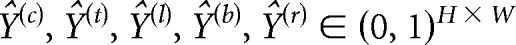. The marked extreme points and corresponding images then needed to be fed into the hourglass network, 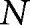. At the same time, we calculated the adjacency spectrum in the training set temporarily stored in ${\text{Spec}}_{0}$ after storing the adjacency spectra of all targets. The cluster radius, 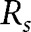, of the adjacency spectrum corresponding to the target needed to be calculated. The second stage consisted of geometric combinations and verification of the adjacency spectrum. In the center-grouping stage, according to ExtractPeak, the heatmap was transformed into the coordinate set 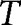, 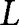, 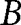, and R of the key points; the distances between the points were calculated and sequentially combined according to the distance. Using the geometric relationship, the combinations of the key point and the center point were verified one by one until the target outlook was formed. After the geometric relationships were combined, the semisuccessful target was temporarily formed, with the adjacency spectrum of the semisuccessful target calculated and verified by the ${\text{Spec}}_{0}$ of the first stage. If the verification was successful, it would eventually form a fully successful target; otherwise, it would continue to combine. The algorithm of GFS-ExtremeNet is shown in the Fig. 7.

### Training detail and index.

We trained our network on the TensorFlow framework (26) with a Tesla K40C and 128-GB memory in an Ubuntu 16.04 system. In the training process, we generally followed the parameters set by the original ExtremeNet model: the learning rate of the network was 0.00025, and the optimizer for the training network was Adam. We fine-tuned our network from a pretrained ExtremeNet model during training. In addition, we used a total of 4 graphics-processing units (GPUs; Tesla K40C) to train 100,000 generations with a batch size of 28.

The average precision (AP) over a set of fixed recall thresholds was used to evaluate the performance of the models. The intersection-over-union (IoU) threshold was set to 0.5 (AP50) and 0.75 (AP75). AP(0.5:0.95) corresponds to the average AP for IoU from 0.5 to 0.95, with a step size of 0.05. For each test image, the network generated five heatmaps and then applied our center-grouping algorithm to these heatmaps. Following ExtremeNet, we kept the original image resolution instead of resizing it to a fixed size.

### Data availability.

The codes and data sets that support the findings of this study are available on https://github.com/jiangdat/GFS-ExtremeNet.
